# Changes in Osseous Morphology Following Non‐Surgical Periodontal Therapy: A Possible Paradigm Shift for the Treatment of Intrabony Defects?

**DOI:** 10.1111/jcpe.14141

**Published:** 2025-03-13

**Authors:** Luigi Nibali, Pierpaolo Cortellini

**Affiliations:** ^1^ Periodontology Unit, Centre for Host Microbiome Interactions, Faculty of Dentistry, Oral & Craniofacial Sciences King's College London London UK; ^2^ Private Practice London UK; ^3^ Private Practice Florence Italy; ^4^ European Research Group on Periodontology (ERGOPERIO) Bern Switzerland; ^5^ Section of Periodontology, Department of Oral Health Sciences KU Leuven & Dentistry, University Hospitals, KU Leuven Leuven Belgium

**Keywords:** bone, non‐surgical, periodontitis

## Abstract

Sub‐gingival non‐surgical periodontal therapy (NSPT) constitutes step 2 of periodontal therapy, aiming to disrupt the subgingival microbial biofilm and resolve or reduce inflammation of the supracrestal periodontal tissues. A plethora of original studies and reviews have been published over several decades, highlighting its fundamental importance in periodontal therapy and in tooth survival. Evidence shows that step 2 therapy leads to probing pocket depth reduction, associated with increase in gingival recession, clinical attachment level gain and improvements in patient‐reported outcome measures, as well as to a reduction in local and, to some extent, systemic inflammatory markers. In cases of intrabony defects, NSPT has traditionally been considered a necessary step to improve periodontal tissue tone and reduce inflammation, thus paving the way for the surgical step, such as periodontal regeneration. This commentary presents compelling arguments for a paradigm shift showing that step 2 therapy also leads to changes in osseous morphology, particularly evident in, but probably not limited to, intrabony defects. This introduces a new dawn, where attempts should be made to understand the mechanisms behind this phenomenon and to maximise the potential of non‐surgical therapy to achieve disease resolution and defect/pocket improvements while reducing patient morbidity even in complex periodontal defects.

## 1 Introduction

The procedure commonly referred to as non‐surgical periodontal therapy (NSPT) is the mainstay of periodontal therapy, as it aims to disrupt the subgingival biofilm and remove calculus from roots of teeth affected by periodontitis, thus re‐creating the conditions for the healing of periodontal lesions. NSPT has been defined under different terms and modalities over the years, such as scaling and root planing, curettage, subgingival debridement, root surface debridement and more recently subgingival instrumentation, step 2 periodontal therapy and professional mechanical plaque removal (PMPR) (Goldman 1930; Aleo et al. 1974; Needleman et al. 2015; Sanz et al. 2020). These different definitions capture different aims and techniques, ranging from removal of soft plaque (debridement), plaque biofilm and calculus (scaling), diseased cementum (root planing) or soft tissue lining of periodontal pockets (curettage). The evolution of NSPT has occurred in parallel with landmark studies that have clarified wound healing mechanisms and have gradually placed emphasis on biofilm disruption more than on aggressive removal of diseased cementum (Echeverria and Caffesse 1983; Moore et al. 1986; Nyman et al. 1988; Mombelli et al. 1995). Minimally‐invasive non‐surgical periodontal therapy is the direct result of these concepts, as it targets calculus removal and biofilm disruption with minimal trauma to the soft tissues, thus promoting healing while minimising gingival recession (Ribeiro et al. 2023). This paper will review current concepts related to NSPT, with a focus on expected healing outcomes.

### 1.1 Wound Healing and Clinical Outcomes Following NSPT

Classic studies have investigated wound healing mechanisms following NSPT. These include sequential phases, from haemostatic to inflammatory, granulation and maturation phases. During this process, the epithelium proliferates on periodontal tissues lining the debrided root surface (1–2 weeks), followed by re‐organisation of collagen bundle fibres, remodelling and maturation of the connective tissue and re‐attachment to the debrided root surface (4–8 weeks) (Waerhaug 1978; Caton and Zander 1979). Thus, it is clear that NSPT results in profound changes to the periodontal pocket environment, which are reflected in macroscopical clinical changes. These could be summarised in changes measurable as:

– clinical parameters
– microbiological outcomes
– local inflammation
– systemic inflammation
– patient‐reported outcomes

A review by Cobb in 2002 highlighted how, although manual, sonic and ultrasonic scalers cannot completely remove all sub‐gingival plaque and calculus, consistent and predictable reductions in probing pocket depths (PPD) and clinical attachment level (CAL) gain are expected following NSPT (Cobb 2002). In particular, initial shallow sites (PPD 4–6 mm) will show a mean PPD reduction of ≈ 1.29 mm and CAL gain of 0.55 mm. Deep sites (PPD ≥ 7 mm) show mean PPD reduction of 2.16 mm and CAL gain of 1.19 mm. Similar results emerge from a more recent European Federation of Periodontology (EFP)‐commissioned systematic review, showing a mean PPD reduction of 1.4 mm at 6–8 months, subdivided into PPD reduction of 1.5 mm for shallow sites and 2.6 mm for deep sites at 3/4 months, with pocket closure (conversion to PPD < 5 mm) of 74%. The average bleeding on probing (BOP) reduction was 57% at 3/4 months (Suvan et al. 2020). It is also interesting to notice that these clinical measurements keep improving up to the 6/8 months re‐evaluation, provided further oral hygiene instructions and maintenance debridement are carried out (Suvan et al. 2020; Paternò Holtzman et al. 2024). These data provide a benchmark for the expected clinical outcomes, which allows assessment of individual patient response to therapy. It is clear that factors such as oral hygiene, smoking habit, obesity, diabetes mellitus and others have effects on these outcomes.

Microbiological outcomes following NSPT were extensively described by Sig Socransky's group in the 1990s and essentially consist of a reduction in the levels of periodontopathogenic bacteria such as *Porphyromonas gingivalis* , *Aggregatibacter actinomycetemcomitans* and *Tannerella forsythia* (Haffajee et al. 1996), with a shift in the composition of the microbial flora towards a more dominant population of Gram‐positive microbes (Cobb 2002). The biofilm disruption and shift in the subgingival microbiota are accompanied by a reduction in local inflammatory markers, measured in gingival crevicular fluid and saliva (de Lima Oliveira et al. 2012; Koidou et al. 2020; Teles et al. 2024), as well as changes in systemic inflammatory parameters (Yue et al. 2020; Orlandi et al. 2022). Such changes include an initial acute‐phase response, with elevation of systemic markers of inflammation such as C‐reactive protein (D'Aiuto et al. 2004), followed by a longer‐term decrease, which might also be associated with improved measures of vascular function (Tonetti et al. 2007).

It has also clearly emerged that, despite the initial acute‐phase response, patients perceive improvements in their oral health‐related quality of life following NSPT (Shanbhag et al. 2012; Botelho et al. 2020).

While all the outcomes above are fairly established and expected, another interesting aspect is emerging and represents the focus of this paper. Can non‐surgical therapy result in morphological changes to the alveolar bone?

### 1.2 Alveolar Bone Changes

Reviews focusing on the outcomes of NSPT (Cobb 2002; Suvan et al. 2020), as well as histological papers describing the healing process (Waerhaug 1978; Caton and Zander 1979), did not mention any alterations to the alveolar bone following NSPT. In fact, it used to be common practice to perform NSPT only as a preparatory step to periodontal surgery in case of infraosseous defects, or in some cases to avoid instrumenting intrabony defects pre‐surgery altogether to avoid excessive soft tissue contraction which might compromise surgical outcomes (Becker and Becker 1993; Browning et al. 2009). It was even suggested that, due to the difficulty of reaching the apical portions of deep pockets with non‐surgical therapy, deep periodontal pockets should be treated with direct vision, that is, after the reflection of conservative flaps (Rateitschak‐Plüss et al. 1992). The principle behind this approach was that the aim of NSPT in intrabony defects was just to reduce tissue inflammation before surgical entry, which was then needed to modify osseous morphology in either a resective or regenerative way, according to indications dictated by osseous anatomy. Landmark studies assessing scaling and root planing or open flap surgery on intrabony defects showed the potential for radiographic osseous gains (Rosling et al. 1976; Renvert et al. 1985). However, this was very limited after non‐surgical approaches, so much so that it was postulated that, as both connective tissue adjacent to the defect wall and inflammatory tissues were not removed non‐surgically, no significant changes in bone levels could be expected (Renvert et al. 1985).

A new wave suggesting that alveolar bone changes could occur after NSPT started a couple of decades ago but has only recently gained momentum. Hwang and co‐workers investigated alveolar bone changes following scaling and root planing using subtraction radiography in 13 patients with 39 intrabony defects (Hwang et al. 2008). They reported an increase in radiographic density by subtraction radiography in 83% of treated areas, particularly in apical portions of the defects. This was followed by a retrospective study by one of the authors of this review, showing for the first time linear radiographic bone changes in 126 intrabony defects amounting to approximately 1‐mm defect reduction at 12 months, with widening of the defect angle (Nibali et al. 2011). A series of studies have since then been published, focusing on minimally‐invasive non‐surgical periodontal therapy (MINST), based on the principles of gentle defect debridement under magnification and minimum trauma to the soft tissues and in particular to the papilla in intrabony defects. MINST studies consistently show that PPD reductions and CAL gain are accompanied by reductions in radiographic defect depth up to 12 months post‐treatment, generally ranging from 1.5 mm to ≈3 mm (Anoixiadou et al. 2022; Mehta et al. 2024; Nibali et al. 2018). It is still unclear whether baseline defect morphology affects differential healing with regard to osseous changes following NSPT (Nibali et al. 2023) and if systemic antibiotics and other factors may influence these results (Nibali et al. 2011). However, we are not aware of any published randomised controlled trials assessing the efficacy of MINST compared with conventional methods for non‐surgical therapy. Case series have also suggested a potential benefit of adjunctive lasers in the non‐surgical treatment of intrabony defects (Al‐Falaki et al. 2016). Although the evidence is still limited and should be systematically assessed, a clear picture is emerging: *non‐surgical periodontal therapy can result in morphological changes to the alveolar bone*.

**FIGURE 1:**
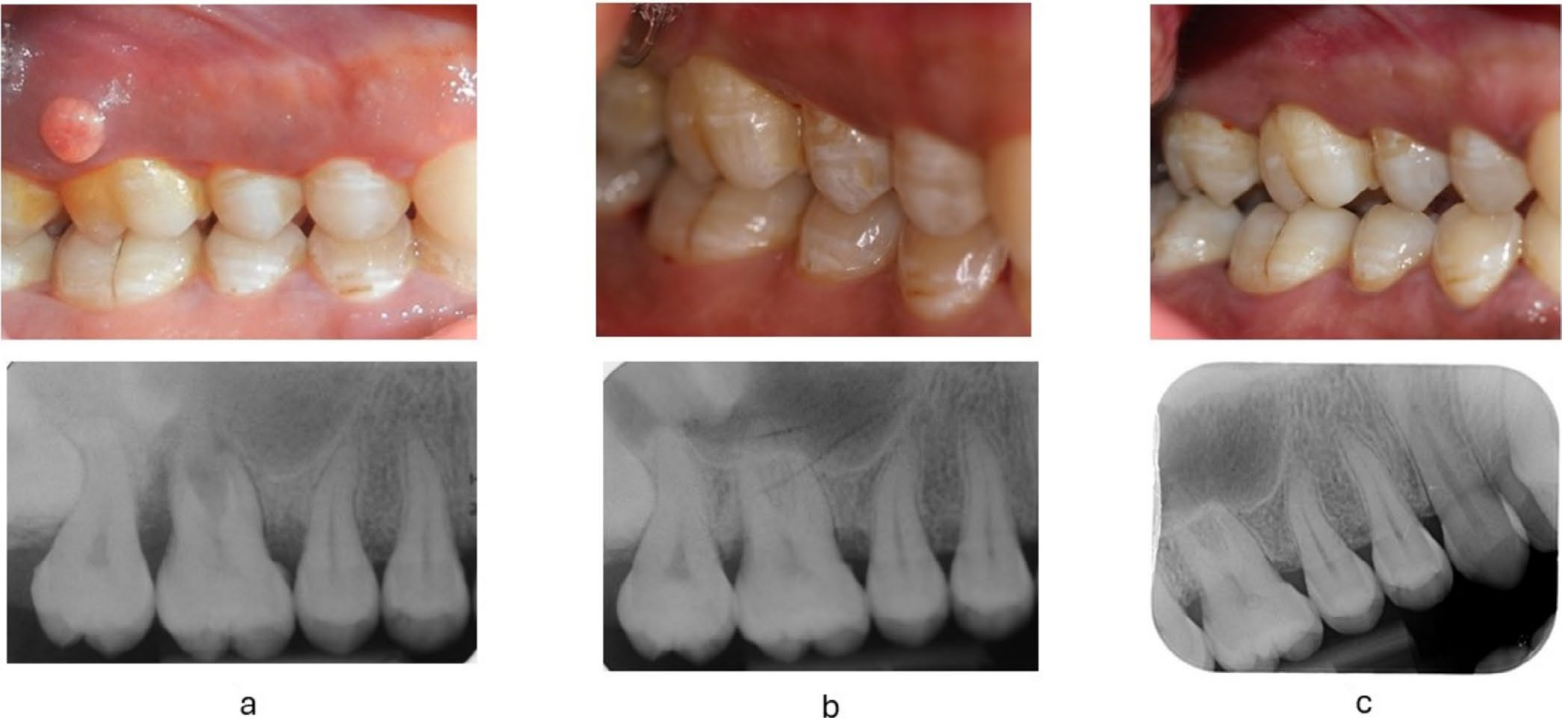
Baseline, 1‐year and 5‐year follow‐up of upper right first molar treated with steps 1 and 2 periodontal therapy, including MINST. The tooth was responsive to sensibility testing at baseline (a) and presented a disto‐buccal PPD of 11 mm coupled with distal furcation involvement degree II. The follow‐ups at 1 year (b) and 5 years (c) show clinical resolution of the pocket (4‐mm pocket depth not bleeding on probing) and furcation (no furcation probing), associated with the closure of the radiographic intrabony defect.

Compared with surgical studies (Nyman et al. 1982), histological studies are still lacking in NSPT. A study using a dental endoscope as an adjunct to subgingival instrumentation showed the potential for bone apposition to occur following NSPT, associated with the removal of subgingival biofilm, formation of a stable blood clot and reduction of chronic inflammation (Wilson et al. 2008). This should question the concepts of ‘periodontal regeneration’, normally defined as functional connection between the augmented soft tissues and alveolar bone to the once infected and morphologically altered root surface. This functional connection and ‘restitutio ad integrum’ are obviously impossible to ascertain if not histologically. However, when extensive PPD reduction and CAL gain occur in conjunction with radiographic bone gain, it is legitimate to wonder what healing process has actually occurred and how different that might be from what is achieved by ‘regenerative/reconstructive’ surgical procedures with the use of biomaterials. This is in line with recent findings of the lack of adjunctive effect of regenerative biomaterials when minimally‐invasive surgical flaps are employed (Aslan et al. 2020; Cortellini et al. 2022). A plausible healing mechanism involves the ability to harness the intrabony defects potential of healing towards repair/regeneration, as measured by high levels of gingival crevicular fluid biomarkers associated with repair, such as vascular endothelial growth factor (VEGF) and osteoprotegerin (OPG) (Santamaria et al. 2023; Koidou et al. 2022). Their temporary increase post‐treatment may trigger healing events leading to regeneration (Koidou et al. 2022), resulting in extensive clinical and radiographic improvements (see example in Figure 1 of a patient treated with MINST and followed up to 5 years later). It is not clear if after NSPT this healing leads to the formation of a long junctional epithelium and new bone apposition/mineralisation or to actual functional connection between new bone and new periodontal ligaments fibres. Histologic evidence of a ‘race’ between epithelium growing apically and periodontal ligament growing coronally (Melcher 1976; Nyman et al. 1982; Sander and Karring 1995) opens a door to the hypothesis of a healing pattern with the formation of long junctional epithelium in the coronal part of the pocket and new bone apposition/mineralisation with functional connection to root cementum through new periodontal ligaments fibres in the apical part.

### 1.3 Implications for Treatment Protocols

Current EFP guidelines stress the importance of steps 1 and 2 of periodontal therapy before reassessment and consideration about whether to advance to surgical therapy (Sanz et al. 2020). An ‘adequate’ amount of time should be waited for following NSPT (step 2), although the exact timing is not very clear and may need to be adapted to each patient and site. Several years ago, a systematic review investigated this aspect, in relation to regenerative surgery of intrabony defects (Nibali et al. 2015). Out of 293 included studies, 10% did not incorporate NSPT in the study protocol before regenerative surgery and 14% did not report about NSPT. Of the remaining 76% of studies, the reassessment time after NSPT was between 2 weeks and 6 months before surgery. No included papers reported clinical and radiographic data before and after NSPT prior to periodontal surgery, showing that the non‐surgical step of therapy was not considered important for the regenerative outcomes (Nibali et al. 2015).

It is indeed clear that, despite good healing after NSPT, a number of sites will still require surgical interventions in order to reach endpoints of therapy (Mehta et al. 2024). The current understanding of a gradual change to osseous architecture of intrabony defects based on the evidence discussed above, suggests that surgical regenerative/reconstructive therapy of intrabony defects should only be conducted 6–12 months following NSPT, in order to allow for the healing to occur and the new osseous architecture to be established first. Here comes a clinical problem in the management of these sites, since a pocket, though of reduced depth, will be still present during the time elapsing from the application of NSPT and the potential bone maturation. These sites need frequent assessments, observation and debridement during the 6–12 months healing period. At this point of ‘late re‐evaluation’, the presence of a residual pocket depth possibly associated with a residual intrabony defect might suggest the indication for the application of regenerative/reconstructive surgery. Therefore, the authors suggest a phased re‐evaluation protocol after step 2 therapy, consisting of:

– review 1 month after step 2, to assess resolution of marginal inflammation and patient compliance (oral hygiene assessment and re‐enforcement);
– re‐evaluation 3 months after step 2 (when most soft tissues healing has occurred), to re‐assess PPD and CAL, as well as oral hygiene and compliance;
– further re‐evaluations 6 and 12 months after step 2 (when most hard tissues healing has occurred), to re‐assess PPD, CAL, oral hygiene, compliance and radiographic osseous changes.

**FIGURE 2:**
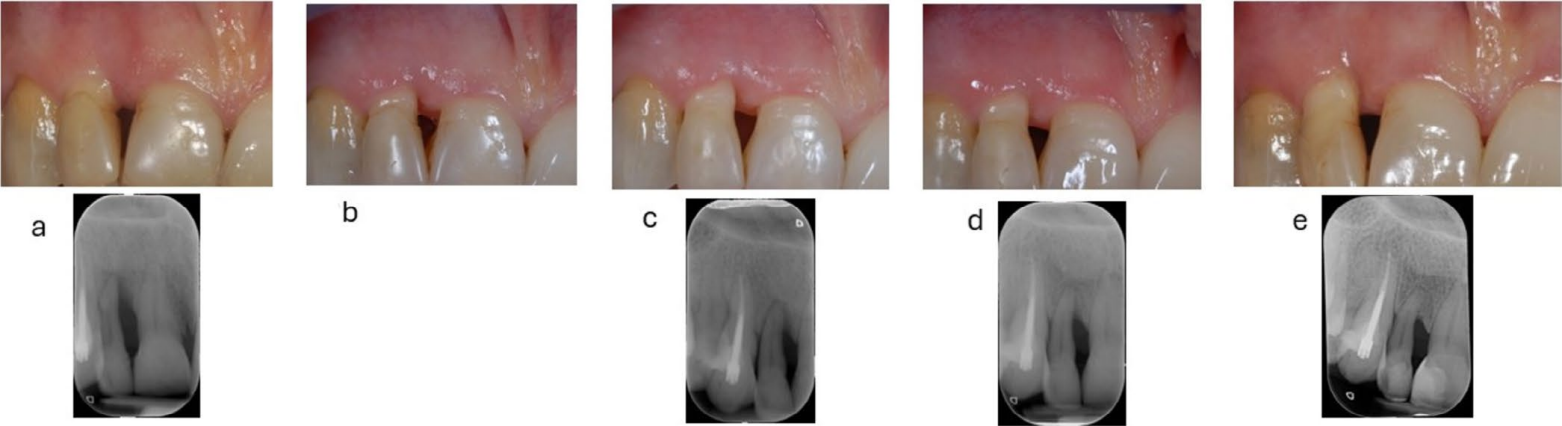
Sequence of images and radiographs showing the healing development following non‐surgical periodontal therapy (NSPT). (a) Baseline condition of a site presenting with inflammation, swelling, exudate and a deep intrabony defect associated with a 10‐mm pocket. This site is amenable to periodontal regeneration. (b) 6 weeks after NSPT at first re‐evaluation it is evident the resolution of gingival inflammation and the contraction of the marginal soft tissues. (c) Second re‐evaluation at 3 months showing further remodelling of the soft tissues. The radiograph shows an initial re‐mineralisation of the intrabony defect associated with 5‐mm pocket depth (PD). (d) Third re‐evaluation at 1 year showing a considerable re‐mineralisation associated with a PD of 4 mm. The clinical condition of the site after NSPT does not indicate the application of regenerative surgery. (e) 10 years follow up with stability of hard and soft tissues.

**FIGURE 3:**
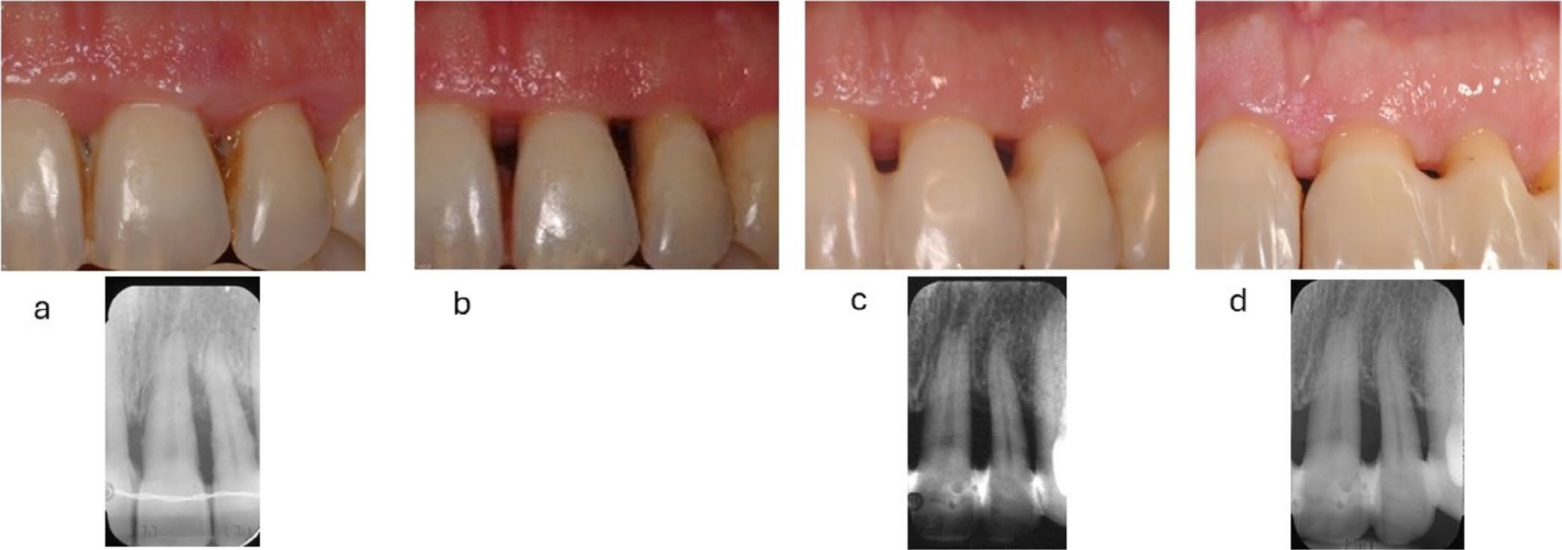
Upper left central incisor at baseline (a) Note the severe inflammation and the deep intrabony defect associated with a 11‐mm PD. This site is amenable for periodontal regeneration. (b) Second re‐evaluation at 3 months showing a noticeable remodelling of the soft tissues. A 5‐mm PD is still detectable. (c) Third re‐evaluation at 1 year, showing the almost complete resolution of the intrabony defect associated with a PD of 4 mm. The clinical condition of the site after NSPT does not indicate the application of regenerative surgery. (d) 15‐year follow‐up showing stability of hard and soft tissues.

**FIGURE 4:**
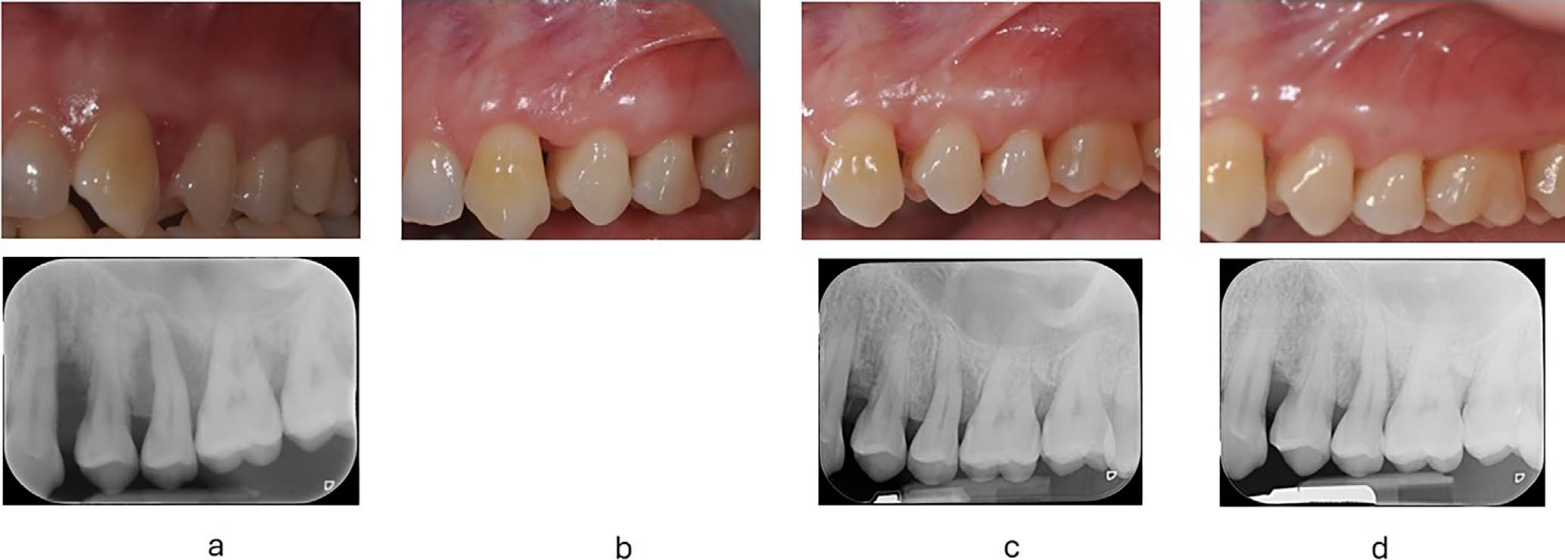
Baseline photograph and periapical radiograph showing very severe inflammation on the upper left sextant (a). Deep intrabony defects associated with deep pocket are evident at the canine (PD 9 mm) and second premolar (PD 12 mm). Both sites are amenable for periodontal regeneration. (b) Second re‐evaluation at 3 months showing the resolution of inflammation and the considerable remodelling of the soft tissues. Residual pockets are still detectable distal of the canine (5 mm) and disto‐palatal of the second premolar (7 mm). (c) Third re‐evaluation at 1 year showing the almost complete resolution of the intrabony defect of the canine (PD 4 mm) and the substantial re‐mineralisation at the second premolar (PD 6 mm). The clinical condition after NSPT does not indicate the application of regenerative surgery at the canine. The premolar will be re‐evaluated during the incoming year. (d) 2‐year follow‐up showing the presence of a residual intrabony component at the second premolar, associated with a 6‐mm pocket. This site will be now treated with periodontal regeneration.

In cases of prevalently intrabony defects and in the absence of clear signs of marginal inflammation, suppuration or patient discomfort, the first re‐evaluation including periodontal probing could be carried out at 6 months rather than 3 months. During these visits, gentle maintenance periodontal debridement (supra‐ and sub‐gingival) is recommended. Clinical judgement is needed to adapt this plan to each specific case, as further interventions such as adjuncts and/or surgical procedures may need to be carried out earlier in some instances. This proposed protocol is illustrated by the cases reported in Figures 2 and 3 showing the healing remodelling of soft and hard tissues during the 1‐year follow‐up that were decisive for the resolution of the periodontal defects, granting long‐term stability of the osseous changes obtained following NSPT. Figure 4 shows a case where regenerative surgery is still indicated in one site after NSPT. Regenerative surgery in this case will be applied to a defect of a completely different and more favourable morphology than at baseline. It could be argued that conventional protocols (NSPT followed by re‐evaluation at 3 months and regenerative periodontal surgery on residual pockets) may achieve the ‘pocket closure’ outcome more quickly. However, these authors feel that the proposed minimally‐invasive protocol may be more acceptable and financially viable for patients, hence leading to favourable outcome in a larger proportion of cases.

In conclusion, this review suggests that alveolar bone changes occur following non‐surgical periodontal therapy, particularly in intrabony defects, warranting reflection over a change in the current clinical protocols, particularly for these types of defects.

## Conflicts of Interest

The authors declare no conflicts of interest.

## Data Availability

Data sharing not applicable to this article as no datasets were generated or analysed during the current study.
